# Eagle syndrome after a fracture of complete ossified stylohyoid ligament from indirect trauma treated using local steroid injection: A case report

**DOI:** 10.1097/MD.0000000000020818

**Published:** 2020-06-19

**Authors:** Yong Won Lee, Jihyun Chung

**Affiliations:** a Department of Otorhinolaryngology-Head and Neck Surgery, Veterans Health Service Daejeon Hospital; b Department of Anesthesiology and Pain medicine, Daejeon St. Mary's Hospital, College of Medicine, The Catholic University of Korea, Daejeon; c Department of Anesthesiology and Pain medicine, College of Medicine, The Catholic University of Korea, Seoul, Republic of Korea.

**Keywords:** Eagle syndrome, fracture, ossification, steroid injection, stylohyoid complex syndrome, stylohyoid ligament

## Abstract

**Rationale:**

Stylohyoid complex syndrome is characterized by various cervicopharyngeal symptoms related to the ossification and abnormality of the styloid process, stylohyoid ligament, and the lesser horn of the hyoid bone. Eagle syndrome is the most well-known of the spectra of these diseases. Although surgical treatment is considered effective, conservative treatment may be beneficial if symptoms arise because of inflammation of the soft tissues attached to the styloid process or hyoid bone.

**Patient concerns:**

A 68-year-old man presented with pain in the right side of the neck and odynophagia after trauma on his philtrum. He was diagnosed with Eagle syndrome elicited by a fracture from indirect trauma. Despite analgesic medication and physiotherapy, the pain had somewhat relieved but persisted for 1 year.

**Diagnosis:**

Computed tomography revealed complete ossification of the bilateral stylohyoid complex. A fracture was observed in the ampulla on the right side of the neck. One year later, the fracture resolved by complete union.

**Interventions:**

Ultrasonography was performed and abnormal ossification was observed on the right side of the neck. Five milligrams of dexamethasone at a concentration of 1 kg/m^3^ was slowly injected into the tender point under ultrasonographic guidance.

**Outcomes:**

The patient reported immediate reduction of pain and was satisfied with the resolution. No recurrence was observed during a 6-month follow-up period.

**Lessons:**

Although traumatic fracture of the ossified ligament elicited the syndrome, the results were satisfactory because the origin of the patient's pain was presumed to arise from inflammatory conditions. This case demonstrates that treatment with local steroid injection may be appropriate for patients who present with pain originating from muscles and ligaments.

## 1 Introduction

Ossification or abnormality of the stylohyoid chain or complex (SHC), comprising the styloid process, stylohyoid ligament, and the lesser horn of the hyoid bone, may cause various cervicopharyngeal symptoms such as dysphagia, dysphonia, odynophagia, headache, and chronic neck pain that may radiate to the ear or eye.^[1–6]^

The nomenclature widely varies for the syndromes associated with SHC. They may be referred to as Eagle syndrome (the most well-known of the syndromes), stylohyoid syndrome, or pseudostylohyoid syndrome depending on the presence or absence of previous surgery or trauma and ossification.^[2]^ However, because the symptoms may arise due to anomalies of either or both the styloid process and stylohyoid ligament, or the absence of variations in ossifications and diverse mechanisms of development, we will use the term stylohyoid complex syndrome to describe the condition most comprehensively.^[3,4]^

The proposed mechanisms of developing symptoms include the following: ossification-induced irritation of the surrounding neurovascular structures, scar tissue, and tightened mucosa post-tonsillectomy, moving across the styloid process; pulled 9^th^ cranial nerve by contracture of the pharyngeal muscle against the fixed ossified SHC; displacement of the ossified part by trauma; reduced elasticity of the SHC; and/or inflammation.^[1–6]^

In particular, tendinitis or tendinosis, one of many relevant inflammatory disorders, is believed to cause cervicofacial pain and tenderness, headache, and otalgia, despite normal ossification of the SHC; this occurs at the muscular attachment site of the styloid process and the lesser horn of the hyoid bone. This condition may be an indication for conservative treatment, such as anti-inflammatory medication or local steroid injection, rather than surgical interventions.^[2,7]^

We report the case of a 68-year-old man with a traumatic fracture of the ossified SHC that was satisfactorily resolved with local steroid injections administered for cervical pain and odynophagia that had persisted after complete healing of the fracture. To the best of our knowledge, these conditions have not been reported thus far in the literature, and the present case will aid in understanding the applications of conservative treatment.

## 2 Case report

A 68-year-old man presented with pain on the right side of the neck and odynophagia that occurred at trauma 7 days before presentation. At that time, the patient had experienced a stairway slip and fall, with his philtrum striking against the edge of the stairs and causing a 3.5 cm laceration of the labial mucosa of the upper lip, which was duly sutured. There was no loss of consciousness and no damage to the teeth.

Despite complete healing of the injury site, there was no improvement in cervical pain and odynophagia. When the patient's mouth was opened >3.1 cm, or when a straw was used for the intake of semifluidic formula, the patient experienced pain on the upper part of the hyoid bone and near the mastoid process; this was not accompanied by a cracking sound. The degree of pain was classified as 6 on the visual analog scale (VAS) of 0 to 10, where 0 equals no pain and 10 equals unbearable pain. There was no discomfort on the left side of the neck and no restriction of horizontal head movement.

During physical examination, firm calcified lesions were palpated in both tonsils, as well as between the hyoid bone and submandibular gland on both sides of the neck. Pain was elicited by pressing the lesions on the right side of the neck, but not by pressure stimulation of the lesions on both tonsils or on the left side of the neck.

The patient was diagnosed with diabetes and hypertension, chronic kidney disease, arrhythmia, heart failure, and gout; he was prescribed medications for each of these conditions. The patient had undergone percutaneous coronary intervention and coil embolization of a cerebral aneurysm 4 months and 6 months before presentation, respectively. There was no history of head and neck surgery.

**Figure 1:**
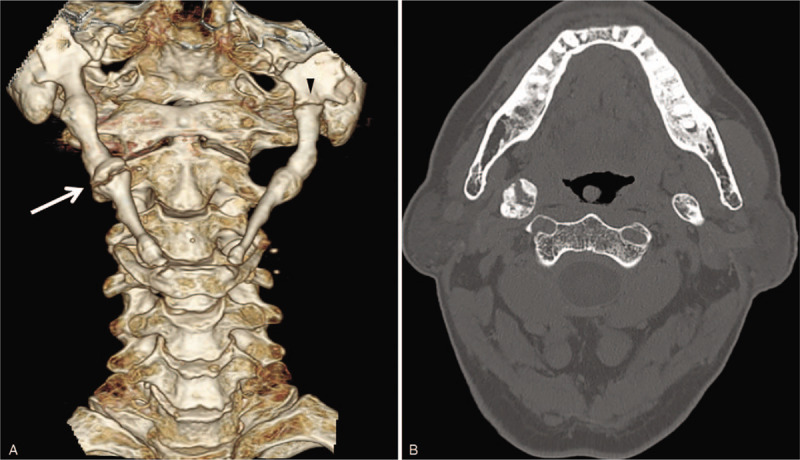
(A) A 3-dimensional computed tomography scan shows bilateral complete stylohyoid complex ossification. A fracture is present on the ampullary area on the right side (arrow) and a gap is seen on the left side of the proximal area (arrowhead). (B) Axial computed tomography demonstrating a fracture on the right ossified stylohyoid ligament.

Computed tomography (CT) revealed complete ossification of the bilateral SHC. A fracture line was observed in the ampullary area on the right side of the middle SHC, and a gap on the left side of the proximal SHC (Fig. 1).

According to these findings, the patient was diagnosed with Eagle syndrome caused by a fracture of the right ossified SHC from indirect trauma. Due to the recent vascular procedures, treatment with medication and physical therapy was decided.

The patient was followed up for 1 year with analgesic treatment, as well as application of heat to the painful area. The patient reported that the pain was slightly relieved with medication or application of a hot pack; however, the pain persisted throughout the follow-up period.

The remaining pain occurred between the hyoid bone and the submandibular gland, radiating to an area on the right side of the mastoid process. The degree of pain was classified as VAS 3; the pain aspect included a feeling of pressing on the throat and pulling of the jaw, which was exacerbated by swallowing, yawning, and opening the mouth wide. Vertical and horizontal movements of the head were not hindered and did not cause pain.

**Figure 2:**
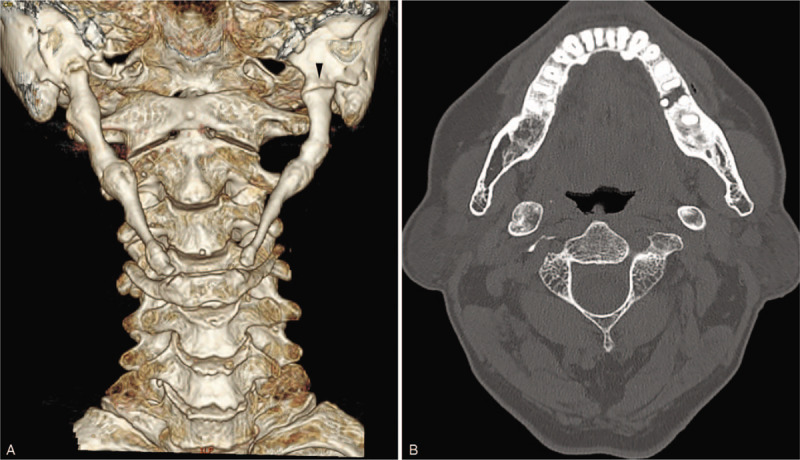
(A and B) Computed tomography scans show complete union at 1 year after trauma. (A) Note the gap on the left side appeared unchanged (arrowhead).

Follow-up CT showed no existing fracture line on the right side of the neck, with a clearer indication of complete recovery than that observed on the previous CT images. The gap of the proximal SHC on the left side, which had not caused any symptoms, remained (Fig. 2).

**Figure 3:**
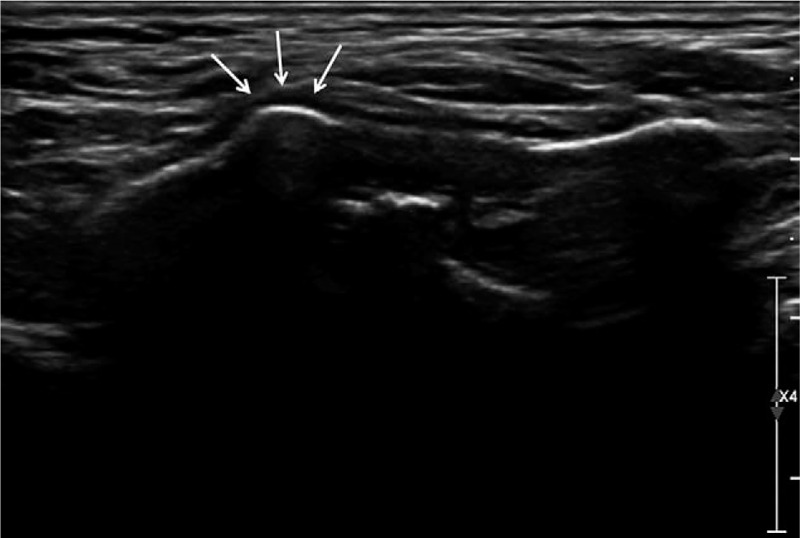
Ultrasonographic finding of right ossified stylohyoid ligament and identified tender point (arrows).

Because conservative treatment was considered more appropriate than surgery, local steroid injection was selected. Ultrasonography was performed and abnormal ossification was observed on the right side of the neck. Tenderness was indicated in the identified area, which corresponded to the ampulla on CT images (Fig. 3). Five milligrams of dexamethasone at a concentration of 1 kg/m^3^ was slowly injected into the tender point under ultrasonographic guidance. The patient reported an immediate reduction of pain, with pain intensity below 1 on the VAS. He was satisfied with the resolution, and showed no recurrence during 6 months of follow-up after dexamethasone injection.

## 3 Discussion

Ossification of the SHC is caused by reactive hyperplasia of the styloid process by irritation, metaplastic change of the ligament stimulated by continuous neck motion or trauma, late ossification of the ligament containing cartilaginous mesenchyme, and degenerative changes of the ligament.^[1,2,8]^ The ossification outcome of the SHC presents with many variations;^[6,9–12]^ it can range from an absence of the styloid process to a complete ossification of the SHC, and may show either a thin and smooth surface or a thick and rough surface. Two or more ossified parts may be segmented or connected by pseudarthrosis. The most common variation known is a prolonged styloid process, although the reported length varies between 2.5 and 4.16 cm. A complete ossification of the SHC is uncommon.

In this case, on the initial CT images at first presentation, it was unclear whether the gap on the left side was a fracture line, or whether it was a gap between ossified segments in the proximal left stylohyoid ligament. However, there were no related symptoms including pain on the left side before and after trauma, and during the follow-up period. Furthermore, it appeared unchanged on the subsequent CT after 1 year, and was located where the tympanohyal and stylohyal portions are supposed to meet. Therefore, we felt it was reasonable to view it as a segmentation, which is one of variants of SHC ossification, rather than a fracture line.

Removal of the ossified portion is considered more effective than conservative treatment for stylohyoid complex syndrome.^[1,3–5]^ However, recent studies have shown that symptoms may occur regardless of the ossification of the SHC: patients with variations of ossification can be symptom-free, patients without styloid process or variations of ossification may be symptomatic, patients with bilateral SHC ossification are often symptomatic unilaterally, and patients who undergo surgery may not respond or may relapse.^[13–15]^

Inflammatory lesions in the muscular and ligamentous attachment areas are a possible cause of symptoms unrelated to ossification.^[2,7]^ They might occur as acute conditions that may be identified at the time of onset, but may persist for >1 year with no identifiable event. A single local steroid injection may resolve short-term symptoms; however, repeated injections might produce satisfactory results with symptoms that persist over a longer period. Hyoid syndrome is a similar disease: tendonitis or tendinosis of muscles attached to the greater horn of the hyoid bone can be treated with triamcinolone injection.^[16]^

Although the present case involved stylohyoid complex syndrome following traumatic fracture of the complete ossified stylohyoid ligament, we suspect several reasons for the response to local steroid injection that may be applied to the inflammatory condition.

First, the fracture was not caused by a direct impact on the neck, but by an impact to the philtrum. The styloid process is a vulnerable site where three muscles, including the stylopharyngeus, styloglossus, stylohyoid muscles, and 2 stylohyoid and stylomandibular ligaments are attached.^[4]^ If a force exceeding the physiological limit is applied, acute stretch or whiplash injury might occur to the muscles or ligaments attached to the styloid process.^[7]^ In addition, fracture might be caused by blunt trauma to the neck, as well as spontaneously without external forces.^[12,17,18]^ Nontraumatic spontaneous fracture, due to intrinsic factors, occurs at weak points in the SHC.^[19,20]^ In this case, a sudden contraction in the muscles and ligaments attached to the region corresponding to the styloid process in response to the trauma might have contributed to causing a fracture, and damage due to stretching of the adherent soft tissue occurring at that time could have caused the syndrome.

Second, the fracture resolved with a complete union. The presence of abundant callus formation and pseudarthrosis was suggested as evidence of the traumatic or nontraumatic fracture of the ossified SHC in the previous reports.^[21–23]^ This is known to be caused by continuous movement of the neck or minor trauma and may cause pain due to a nonunion or displacement. However, the pain persisted in this case, despite complete union with a smooth surface lacking callous formation or pseudarthroses. The patient exhibited asymptomatic ossification of the SHC before trauma, and fracture after the trauma was fully resolved. Therefore, it is assumed that the underlying cause of the pain was not in the ossified SHC, but in the surrounding soft tissues.

In addition, the tender point was obvious and could be confirmed by ultrasonography. Regarding the pain in the peristyloid area, the styloid process and surrounding structures are visualized with ultrasound and injecting at the precise location has great effect on the resolution.^[7,24]^ In this case, the pain had developed in an area thicker than the other part, so it was relatively easy to identify. However, considering that the muscles are mainly attached proximally to the styloid process and the ligaments are attached distally, a part of the muscles and ligaments attached around the tip of the styloid process were presumably the site of origin of the pain.

The present case of stylohyoid complex syndrome was caused by traumatic fracture followed by complete ossification of the SHC. The origin of the patient's pain was presumed to be the muscles and ligaments, and treatment with local steroid injection was satisfactory. Although surgical treatment is considered more effective than conservative treatment for stylohyoid complex syndrome, this case demonstrates that treatment with local steroid injection at the precise location of pain under ultrasound guidance may be worthwhile in patients who present with pain originating from muscles/ligaments or due to inflammatory conditions, before surgical interventions.

## Author contributions

**Conceptualization:** Jihyun Chung.

**Data curation:** Yong Won Lee.

**Writing – original draft:** Yong Won Lee.

**Writing – review & editing:** Jihyun Chung.
